# Cine-EPI can be used to detect coronary artery stenoses in canines

**DOI:** 10.1186/1532-429X-11-S1-P106

**Published:** 2009-01-28

**Authors:** Jordin D Green, Matthias Voehringer, Jacqueline A Flewitt, Sven Zuehlsdorff, John V Tyberg, Matthias G Friedrich

**Affiliations:** 1 Siemens Healthcare, Calgary, AB Canada; 2 University of Calgary, Calgary, AB Canada; 3 Siemens Medical Solutions USA, Inc., Chicago, IL USA

**Keywords:** Fractional Flow Reserve, Blood Oxygen Level Dependent, Echo Planar Imaging, Adenosine Infusion, Late Enhancement

## Introduction

Non-invasive assessment of myocardial ischemia is challenging. Because the BOLD (Blood Oxygen Level Dependent) effect mainly relies on endogenous contrast to differentiate ischemic from non-ischemic tissue, BOLD has the potential to directly assess myocardial oxygenation.

Though T_2_*-weighting is easily achieved using triggered, mid-diastolic echo planar imaging (EPI), it can be sensitive to artifacts. However, by using a cine-EPI approach, it may be possible to use a lower effective TE (TE_eff_), thereby reducing artifacts but maintaining BOLD sensitivity by averaging several mid-diastolic phases during analysis.

## Purpose

To demonstrate that a cine-EPI sequence can be used to detect adenosine-induced oxygenation changes in a stenosis dog model.

## Methods

We developed a cine EPI sequence with the goal of obtaining strong BOLD-weighted imaging but without significant image artifacts. Cine phases were acquired using prospective ECG-triggering, a breath hold (~12 s), a short echo train (4 echoes) with a segmented approach (8 lines/segment), and a bipolar readout. All studies were performed on a 1.5 T MAGNETOM Avanto (Siemens Healthcare, Germany) in canines (n = 4) with a balloon catheter fluoroscopically guided into the left circumflex or left anterior descending coronary artery, to create stenoses as validated by simultaneous fractional flow reserve.

We obtained cine-EPI images in a mid-ventricular slice, before and during an adenosine infusion of 140 μg/kg for two minutes. We repeated this process twice, once with a mid-grade stenosis and once with a high-grade stenosis. At the end of the study, we injected gadolinium (Gd) via our intracoronary catheter and ran a perfusion scan to verify the territory affected by the stenosed artery. We then injected Gd intravenously and performed late enhancement (LE) to verify the absence of infarct. Sequence parameters for cine-EPI: FOV = 300 × 300 mm^2^; matrix = 123 × 128; thickness = 10 mm; temporal resolution = 49 ms; flip angle = 15°; TE_eff_ = 15 ms.

We analyzed the data using a clinically validated software package (cmr^42^, Circle Cardiovascular Imaging Inc., Canada). We used the intracoronary perfusion images to identify the affected and remote myocardium. By averaging 4 phases corresponding to mid-diastole, we measured mean signal intensity (meanSI) in the affected and remote territory during no stenosis and the two stenosis levels, when the subject was at rest and during adenosine infusion. For each territory, we calculated the adenosine response as a percent change in image meanSI going from rest to stress (%ΔSI_aden_) for each of the three stenosis levels (no stenosis, mid-grade stenosis, high-grade stenosis). We then compared %ΔSI_aden_ in a particular territory for the different stenosis levels using a matched pairs t-test (α = 0.05).

## Results

**Figure 1:**
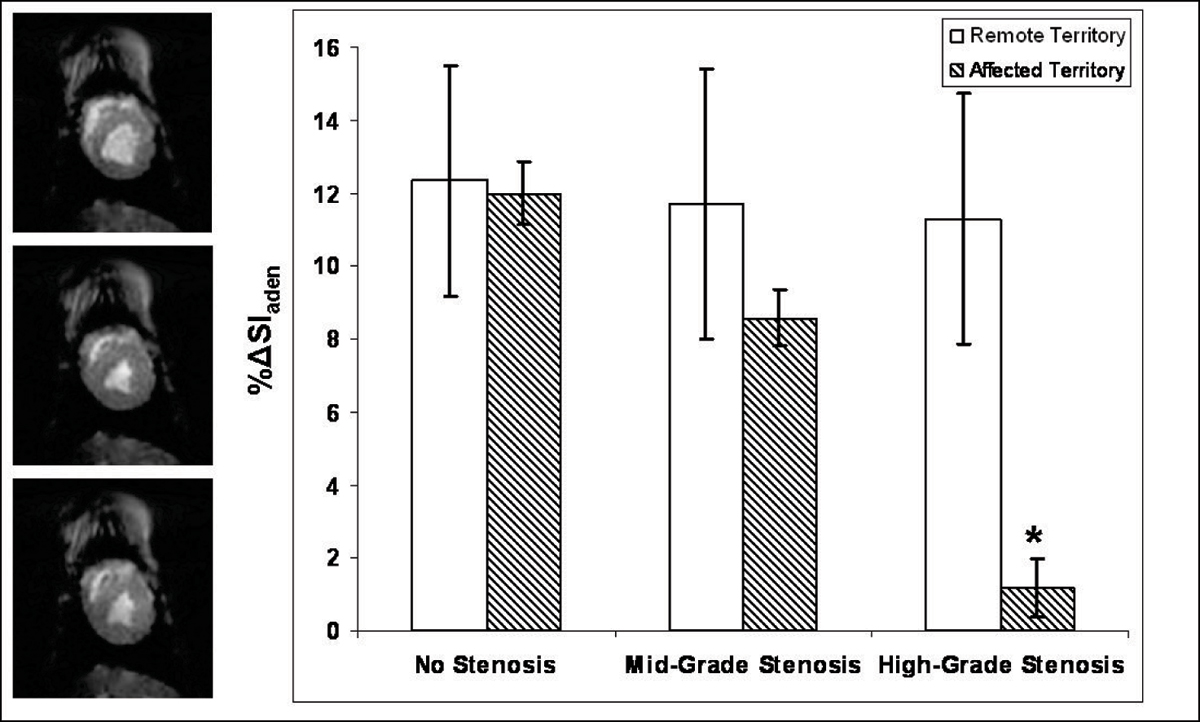
***Left*** **: Typical stress images from one study during the high-grade stenosis, taken at various points in the cardiac cycle using cine-EPI**. *Right*: Plot of %ΔSI_aden_ (%SI change from rest during adenosine infusion) for the affected territory and the remote territory for the three levels of coronary artery stenosis. Error bars represent ± 1 SE. An * indicates statistically significant difference (p < 0.05).

The results are summarized in the Figure 1. In the remote territory, mean %ΔSI_aden_ at no, mid-grade, and high-grade stenosis (± standard error) was 12.3% ± 3%, 11.7% ± 4%, and 11.3% ± 3% respectively. In the affected territory, the same measurements were 12.0% ± 1%, 8.6% ± 1%, and 1.2% ± 1%. Looking at each stenosis level, there was only a statistically significant difference between the two territories for the high-grade stenosis. Looking at each territory, there was no statistically significant difference between the %ΔSI_aden_ observed for the different stenosis levels in the remote territory. For the affected myocardial territory, there was a statistically significant decrease in %ΔSI_aden_ going from baseline to either stenosis level, as well as going from mid- to high-grade stenosis. No LE was observed.

## Conclusion

We have shown that cine-EPI can accurately detect changes in adenosine response in myocardium affected by a high-grade stenosis. We could use a lower TE_eff_ than previously reported for EPI cardiac BOLD because we were able to signal average over several cardiac phases, which in turn reduced image artifacts. Cine-EPI shows promise for identifying regions of ischemia in CMR, simultaneous to functional assessment.

